# Privacy-preserving cancer type prediction with homomorphic encryption

**DOI:** 10.1038/s41598-023-28481-8

**Published:** 2023-01-30

**Authors:** Esha Sarkar, Eduardo Chielle, Gamze Gursoy, Leo Chen, Mark Gerstein, Michail Maniatakos

**Affiliations:** 1 Tandon School of Engineering, New York University, Brooklyn, NY 11201 USA; 2 Center for Cyber Security, New York University Abu Dhabi, Abu Dhabi, 129188 UAE; 3 Program in Computational Biology and Bioinformatics, Yale University, New Haven, CT 06520 USA; 4 Department of Computer Science, Yale University, New Haven, CT 06520 USA

**Keywords:** Computational biology and bioinformatics, Diseases

## Abstract

Cancer genomics tailors diagnosis and treatment based on an individual’s genetic information and is the crux of precision medicine. However, analysis and maintenance of high volume of genetic mutation data to build a machine learning (ML) model to predict the cancer type is a computationally expensive task and is often outsourced to powerful cloud servers, raising critical privacy concerns for patients’ data. Homomorphic encryption (HE) enables computation on encrypted data, thus, providing cryptographic guarantees to protect privacy. But restrictive overheads of encrypted computation deter its usage. In this work, we explore the challenges of privacy preserving cancer type prediction using a dataset consisting of more than 2 million genetic mutations from 2713 patients for several cancer types by building a highly accurate ML model and then implementing its privacy preserving version in HE. Our solution for cancer type inference encodes somatic mutations based on their impact on the cancer genomes into the feature space and then uses statistical tests for feature selection. We propose a fast matrix multiplication algorithm for HE-based model. Our final model achieves 0.98 micro-average area under curve improving accuracy from 70.08 to 83.61% , being 550 times faster than the standard matrix multiplication-based privacy-preserving models. Our tool can be found at https://github.com/momalab/octal-candet.

## Introduction

Precision medicine, the process of tailoring diagnosis and treatment for an individual patient, has far-reaching results when analyzing complex diseases^1^. One of the biggest components of precision medicine is to incorporate patients’ genetic information to the diagnosis, treatment, and decision making^2^. This is further bolstered with the availability of genetic data with the decreasing sequencing costs. In the context of precision medicine for cancer, distinguishability between genetic mutations of normal and malignant tissues is the crux of cancer genomics. These somatic genetic changes accumulated during a person’s life are heavily correlated to the progression of several cancer cases^3^. Somatic single-nucleotide variation (SNV) and copy-number variation (CNV) on protein-coding genes, especially on oncogenes, tumor suppressors and cell cycle regulators are known to cause tumor formation and progress. However, the heterogeneity in various levels makes it difficult to understand precisely which gene is involved in which cancer type. From a data analysis perspective, a statistical inference of cancer type requires analyzing huge volume of genomic data to find correlations between somatic mutations and cancer type.

Machine learning (ML), has had an unprecedented success in correlating complex data patterns to an inference in several critical infrastructures, including healthcare^4^. An ML model is a function (combination of linear and non-linear functions) with trainable parameters which *learns* about the data by decreasing the loss between prediction and true values. This computationally expensive task of training is often beyond the capabilities of personal machines and is outsourced to powerful servers. After a model is trained, it is hosted in the cloud where different parties can use it as a service to predict cancer type of new patients.

There are privacy concerns in using genetic information for this type of inference. Genetic information, especially in the clinical settings, are subject to privacy protections and may not be used in public server and cloud computing setting due to the restriction. A genomic data leakage may also be permanent, unlike other private data like passwords, credit cards, etc., which can be changed. A partial leak of genomic data may reveal important information about the individual and may also be used to reconstruct their genome^5^. If the sensitive data is always encrypted, then it can be protected during computation as well as in transit. In other words, if an entity (like the cloud) is not trusted, data should not appear in plaintext form.

Homomorphic encryption (HE) is a cryptographic technique that allows for computation on encrypted data. In this setting, researchers can use their homomorphically encrypted ML models in public servers without revealing the input, interactions with the input and output, as well as the outputs of the models. The first Fully HE (FHE) scheme for arbitrary computation proposed by Gentry et al.^6^ had prohibitive computational overheads for real-world applications like ML-based inference. During inference an ML model performs linear operations, typically matrix multiplications and additions between the data matrix, the weight matrix, the bias matrix, and non-linear operation on the resultant output. Linear operations in HE become more and more expensive as the number of features increase, i.e. as the amount of information in a dataset increases, the number of operations in HE increases, making encrypted computation impractical. Genomic datasets are inherently high-dimensional and the current privacy-preserving computation literature is limited in exploring these datasets^7,8^. Hence, to effectively compute on genomic data in the encrypted domain, we explore feature engineering methodologies to reduce overhead and improve the inference methodology to increase scalability in the encrypted domain. Our data consists of more than 2 million CNV and SNV information of 11 different cancer types^9^. Using an ML model on the raw cancer dataset would require multiplication of matrices with millions of features (columns), which is extremely expensive in HE.

The intuitive solution towards faster private inference is to reduce the number of computations. This translates to reducing the number of features using feature selection, as can be seen in other HE applications in genomics^10,11^. For dimensionality reduction, we develop a somatic mutation encoding-based feature engineering methodology involving feature (gene) selection and genetic (mutation) information encoding, leveraging both biological intuition and statistical tests. Trivially using just statistical scores may result in overfitting especially for genomic datasets with the number of predictors (*f*) several times larger than the number of samples (|*X*|) i.e. $f>>|X|$, a problem extensively discussed by scientists^12^. Using our methodology of somatic mutation encoding, we reduce the dimensionality of the task (from over 2 million mutations to 43K features), but still the genomic data remains high-dimensional as compared to benchmark ML datasets used for evaluating homomorphic encryption-based privacy-preserving studies. Since our application *needs* several thousands of features for accurate predictions, not only our time budget is completely exhausted by the linear operation, but also standard matrix multiplication does not offer the performance needed.

Another drawback of current HE-based implementations of private inference is that they are designed to maximize throughput, computing on thousands of inputs together to improve efficiency in a cumulative way. However, they suffer in latency, i.e. the algorithms would take the same time to compute on just one input as it would take for thousands of inputs. Therefore, for algorithms that do not optimize for latency would generally wait for thousands of test data points to send a single query to the cloud for the inference on those data points. But the real-world application we consider in this work benefits from improved latency, i.e. it is important for the private inference algorithm to infer on a single data point instead of waiting for a batch of thousands of patients to utilize the query limit. In summary, to enable practical real-world private inference, we need the ability to compute on high-dimensional data in the encrypted domain with low latency and high throughput.

### Threat model

In this work we focus on privacy-preserving inference following the *honest but curious/semi-honest* threat model explored in the cryptography literature^13^. In this work we focus on privacy-preserving inference following the honest but curious/semi-honest threat model. The goal of such an adversary is to gain (sensitive) information while not hindering the computation at the cloud. In our application, we represent the training data accessible to the cloud as ($D_{train}$, $y_{train}$) as training genomic data and labels, respectively. We represent the new patient data as ($D_{patient}$, $y_{patient}$) as the patient’s genomic data and predicted tumor type respectively. In our threat model, an adversary honestly computes $y_{patient}$ but is curious about highly sensitive $D_{patient}$ and $y_{patient}$. Hence, we resort to homomorphic encryption to keep $D_{patient}$ and $y_{patient}$ encrypted. The following points out the objectives/characteristics of the Cloud server and the Client:

1. *Cloud server* The cloud is *honest but curious* i.e. it infers cancer type correctly but is curious of the highly identifying patient (genomic) data motivating encrypted computation at the cloud. The cloud, however, has access to public training data collected from several research entities (like in the case of TCGA which catalogues genomic information, prognosis, diagnosis, on top of personal information like age, gender, race, and ethnicity of the individuals who are identified as case numbers). The resource heavy data collection and maintenance and model training and maintenance are outsourced to the cloud.
2. *Client* The client (may be a hospital or an individual patient) owns sensitive data and wants to perform cancer prediction on the data without revealing the data to the cloud. This data, in our study, is represented by test data.

In a nutshell, for a private cancer detection solution to be practicable, it must have three properties: (1) comprehensive high-dimensional genomic analysis for high detection accuracy with a focus on explainability, (2) cryptographically secure privacy guarantees, (3) practical inference time and high throughput. In developing the privacy-preserving tumor prediction model, we list our contributions as follows:

1. We develop a three-step feature engineering methodology targeted towards practicable encrypted cancer prediction and demonstrate the importance of feature selection and encoding based on biological significance of genetic alterations and statistical tests. We discuss the *predictive genes* from our findings and compare it to Gene Ontology enrichment analysis to understand the significance of certain genes in predicting a cancer type.
2. We build high-performing ML model (for all labels), and binary models using the same encoding for each cancer type. We compare our methodology with the reported baseline and we achieve a 13% increase in accuracy^14,15^.
3. We propose a fast matrix multiplication algorithm for high-dimensional matrices, specifically designed to implement HE-based privacy-preserving logistic regression model to ensure high performance.
4. We optimize our private cancer prediction methodology for low latency as well as high throughput.
5. We implement our privacy-preserving cancer-prediction model using BFV^16^, an FHE scheme, and demonstrate that we can perform privacy-preserving cancer prediction for $\approx$ 500 individuals under 1 min and a single prediction in approximately 1 second. We compare the performance of our privacy-preserving model with an ML model implemented using standard matrix multiplication.
6. We open-source our methodology and implementation.

Paper roadmap: We first discuss data encoding schemes and feature selection using biological intuition and statistical tests in “Somatic mutation encoding” section. Using the selected features, we perform a grid search over several ML models for the best performing model in ”Model selection for cancer prediction” section. We propose our matrix multiplication algorithm for the HE-based implementation of ML model in “Matrix multiplication algorithm” section. We evaluate our final model using accuracy, auc score, f1-score, precision, recall metrics for cancer prediction performance, and computational cost, latency, and throughput for encrypted cancer prediction performance in “Results” section.

## Methods

Since computations in the encrypted domain are expensive, private inference on any type of should prioritize towards less number of computations. This translates to a low number of features and smaller ML models (for example, SVM or logistic regression instead of deep networks). Our methodology can be divided into two parts. In the first part of our methodology (“Somatic mutation encoding” section), we focus on making our dataset compact encoding mutations and reducing the number of features with biological intuitions and statistical tests. This reduces the number of features from over 2 million to 43 K. In the second part of our methodology (“Privacy-preserving cancer inference” section), we propose a matrix multiplication algorithm, particularly catered towards implementing a faster version of privacy-preserving logistic regression-based cancer inference.

### Somatic mutation encoding

For the cancer prediction to be correlated to both CNV and SNV information, the CNV and SNV features can be concatenated together, which can be used to train an ML model. CNV subset has 25 K features. But the SNV subset corresponds to over 2 million mutation rows, which may equate to over 2 million features if each of these mutations is analyzed separately. The concatenated dataset, thus, consists of more than 2 million features. Therefore, instead of representing a mutation as a feature, we represent a gene as a feature with encoded mutation as the value of that feature. However, this approach faces the challenge of compacting mutation information of over 2 million data-points to 25 K data-points (corresponding to 25 K genes).

#### Step 1: gene/feature selection using SNV frequency

Previous studies ^14,15^ on cancer detection using somatic mutations observed that the frequency of mutation of a gene correlates to higher prediction accuracy. We first choose the genes with the highest number of mutations as we hypothesize that there might be an additive effect of the mutations and more somatic mutations will have higher impact on the function of the gene^17^. We then focus on the mutations with high frequency as recurrent somatic mutations have more statistical power to predict a cancer type due to more patients having them. It is also suggested that recurrent mutations are more informative of the mutational processes in cancer, which might help with the prediction of the cancer types^18^ if different types are governed by different mutational processes. Therefore, as the first step of feature selection, we choose genes with higher number of mutations. But each cancer type corresponds to a higher mutation in a different gene. We first rank the genes based on their SNV frequency in the patient cohort for each cancer type by also taking into consideration genes with more than one SNVs on them. We then combine the ranked genes from different cancer types and finally select the top 10,000 genes with highest SNV frequency for each cancer type as our features. This step also ensures removal of genes with low SNV frequency and reduce the dimensionality of our feature space. With all cancer types combined, we have a total of 18,606 genes.

#### Step 2: encoding scheme

**Figure 1:**
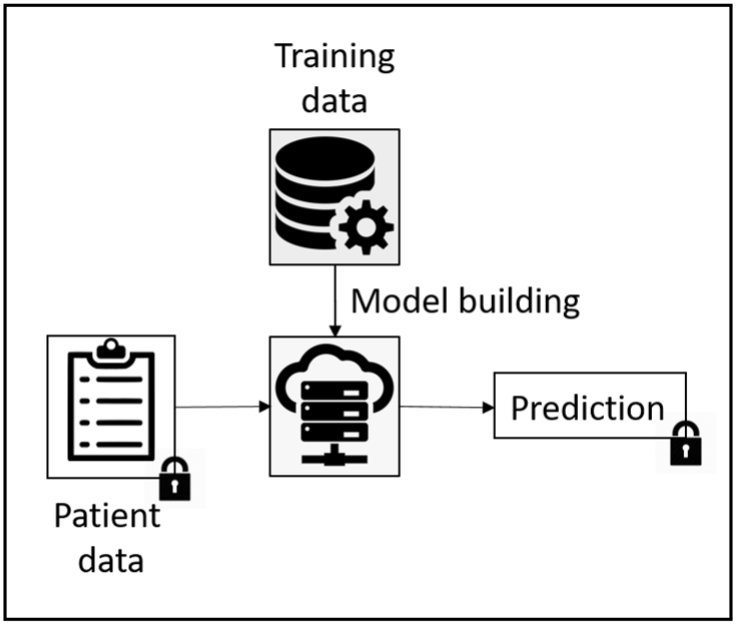
Our threat model for private inference.

Encoding techniques, both for the feature vectors and the target variables, ease in training towards better accuracy. Efficient encoding techniques have been proven to result in better performance in genomic classification tasks as well^19^. As mentioned in the dataset section, each SNV on a gene is represented by multiple characteristics. This information need to be meaningfully merged with the CNV information of each gene. We explore the following encoding schemes based on a biological intuition as explained below:

1. *Using presence of an SNV on a gene* The genes selected using frequency are merged for all cancer types. In this encoding we aim to study if a particular gene (mutation) is highly correlated to a cancer type. We assign a binary value [0, 1] to each of genes of a patient to denote the presence or absence of one or more SNVs. This allows us to encode SNV information in a categorical manner. This binary value per gene is used as a feature.
2. *Using the type and impact of an SNV* The impact of mutation of a gene is calculated using Ensembl Variant Effect Predictor (VEP) (see Supplemantary Information)^20^.
  For each SNV in the dataset, we have two types of measure: 1—a qualitative measure indicating whether an SNV has a tolerated or deleterious effect; 2—a quantitative real-value measure ($s_{i,j}$) representing the strength of the impact and the qualitative confidence with which the mutation can be attributed to the diagnosis. $s_{i,j}$ is the strength of the $i{\text {th}}$ SNV in patient *j*. Each of the encoding scheme are given equidistant real values between 0 to 1. We also experimented with different ranges but did not find any improvement in test accuracy. We think that both of these measures are useful in classifying the tumor type. We first encode the first measure by assigning values for [deleterious, deleterious (low confidence), tolerated (low confidence), tolerated] as [1.0, 0.75, 0.5, 0.25] for each $m_{i,j}$. The reason for this encoding is that a detrimental effect is given the highest value in cancer prediction and similarly a tolerated effect is given the lowest feature value. Either of the effects, when estimated with lower confidence are given lower effect. *m* is the effect of the $i{\text {th}}$ SNV in patient *j*. We then combine this encoding with the second measure as $m_{i,j} \times s_{i,j}$. The final effect values of SNV impact of a gene is the summation of the impacts of all the SNVs on that gene, if there is more than one SNVs. The resulting value per gene is used as a feature.
  In addition to the strength (*s*) of an SNV, the qualitative confidence of the effect of an SNV on a gene is also as a categorical variable with values as [high, moderate, modifier, low]. We encode them as [1, 0.4, 0.7, 0.1] since intuitively we want to assign a higher importance to a high mutation effect. A gene with no mutation is assigned 0 effect. Similar to the previous feature, for a gene with multiple SNVs at different locations, the values are added to finally represent the effective value of all the SNVs in a gene. The resulting value per gene is used as a feature (Fig. 1).
3. *Using CNV of a gene* CNV of a gene is represented as integers between − 2 and 2, indicating if both, one, or no copy of the gene is deleted or duplicated. We scale these values to integers between 0 and 4 as statistical feature selection methods (for example $\chi ^2$ test) often require positive values. Similarly, the resulting value per gene is used as a feature.

For each patient, 18,606 genes with their associated SNV encoding are concatenated with 25,128 genes with their CNV information. In total, we have 43,734 *features* which undergo the following statistical tests.

#### Step 3: feature selection using $\chi ^2$ test

The previous steps of feature selection incorporate biological intuition. In this step, we explore statistical tests for evaluating feature importance. Statistical methods like $\chi ^2$ test do not only inform the feature importance but also help reduce dimensionality of the feature space enabling faster computation (both during training and inference). It has previously been used in genetic information based disease prediction studies^21^. We performed three statistical tests for feature selection after step 2: (1) mutual information, which measures the gain in information or reduction in entropy if a feature is selected, (2) chi-square test, which measures the difference between the actual and the expected output if a feature is selected, and (3) f-score, which measures the Analysis of Variance (ANOVA). We choose chi-square test as a feature selection metric since we achieve the best possible accuracy when compared to feature selection with mutual information or f-score statistics. We choose $\chi ^2$ test as a feature selection metric since we achieve the best possible accuracy when compared to feature selection with mutual information or f-score statistics (as reported in “Results” section). To decide if a feature is independent of the target label (i.e type of cancer), we perform the $\chi ^2$ test where we calculate $\chi ^2$ value of each feature with respect to the target variable. The $\chi ^2$ value of a feature is given as $\sum \frac{(O_i-E_i)^2}{E_i}$ where *E* represents the expected value, *O* represents the actual output and *i* represents each instance of a $\chi ^2$ test between a feature and a target. Note here that, the expected values of a variable is calculated using the distribution of feature values.

We run the $\chi ^2$ test on all genes (CNV and SNV concatenated together) and sort the features in decreasing order of $\chi ^2$ values. The top *n* features are selected and are used to train a classification model. We also use this step to analyze the selected genes and their relative *importance* in cancer type prediction in “Predictive genes” section.

### Privacy-preserving cancer inference

#### Model selection for cancer prediction

While our encoding scheme and feature selection methodologies are targeted towards reducing the number of computations in the encrypted domain, we follow a similar philosophy to select our ML model. We perform a grid search over several small ML models like Support Vector Machine (SVM) (with radial basis function, polynomial and linear kernels), logistic regression, and Deep Neural Networks (DNNs with two fully connected hidden layers with relu activation) as possible classification models (see Supplemantary Information). To capture the non-linearity in the data, we train these models with their respective non-linear activation functions. However, after model selection, we implement the privacy preserving model using the approximation later discussed in “Approximation of non-linear function in private inference” section. We start with the top 1000 features selected by $\chi ^2$ test and increase the number of features by 1000 in each iteration. In our search for best-performing model, we train models using different number of features, different statistical tests for feature selection, different kernels (if applicable), with several regularization techniques, and with different optimization techniques cross-validated over fivefolds. Although we train all the models referring to our grid search, we only evaluate and report the best performing models under each category of model and features in “Results” section.

##### Measures to reduce overfitting

We find that the logistic regression model performs best for several encoding schemes with the best test accuracy of 83.61%. To tackle overfitting, we take measures in two stages: (a) during training, (b) after training. We introduce a Lasso (*l*1) penalty^22^ to the logistic loss function during training such that the features that are unlikely to contribute to the prediction are penalized and weighted zero. Therefore, if the logistic loss function is given by $L(\beta _j)$ where $\beta _j$ represents the coefficients of the features, the loss function after Lasso penalty in the Lagrangian form becomes $L(\beta _j) - \lambda \sum _j^n|\beta _j|$ which is minimized during training. Further, we split the training process into $k=5$ folds such that in every training iteration, the data is sampled differently, i.e. the model is iterated over a slightly different data when training for each split, thus using k-cross validation during training. We also aim for models with higher test accuracy to minimize the difference between training and test accuracy, which is an indicator of overfitting. In the post-training stage we analyze our predictive genes using gene ontology study to interpret cancer prediction with our model and to detect overfitting (if any) of some labels to some genes.

##### Metrics to detect problems of unbalanced data

High test accuracy on unbalanced datasets (with a higher percentage of samples from a particular label) can give a false sense of performance as a random guess (of the label with the highest number of samples) may also result in a high accuracy. For a holistic performance evaluation of our classifiers, we plot Receiver Operating Characteristics (ROC) Curve and report the individual area under curve for each class and the Micro-average Area Under Curve (MAUC) for the classifier. Since ROC curves reflects the entire range of probability threshold, it is a more robust metric and is used in genetic analysis.

##### Binary models

We also build models to predict each cancer type separately (like specific models in Ref.^15^). Note that training of individual classifiers is to show the effectiveness of the gene selection and encoding schemes. Since the sample sizes for training individual classifiers are relatively low, the performance may not translate to larger, more diverse datasets. These models are supplementary to our main prediction model and focus on one type of cancer. These binary models can also serve as independent models since genomic datasets are not balanced. The features for this single cancer type model are chosen following the steps described above and the classifier is trained using a binary label: 0 for the all of the other cancer types and 1 for the cancer type of interest. We create separate models for each of the 11 cancer types and call these models as binary models since the prediction is converted into a binary classification task.

It should be noted here that the *binary* classification ability represented by the ROC curves of individual diseases (in our main prediction model) and the binary models for individual diseases are different because of the feature selection steps. In our binary models, the genes important to a specific disease are selected. However, for our main prediction model, the genes which are cumulatively important for all the 11 labels, are selected. Hence, we report both the analyses in “Results” section.

From analyzing the nature of genomic mutation data and the trends in accuracy (details in “Results” section), we observe that regardless of the ML model selected, the matrix multiplication would involve high-dimensional matrices (Dot product between weights and several thousands of features is common for all the ML models explored in our work). Therefore, following standard matrix multiplications would require a large number of multiplications corresponding to this high-dimensional dataset. The private cancer prediction methodology is characterized by a private inference protocol proposed in “Private inference protocol” section and then the fast matrix multiplication methodology, crux of the private ML algorithm, is proposed in “Matrix multiplication algorithm” section.

#### Private inference protocol

**Figure 2:**
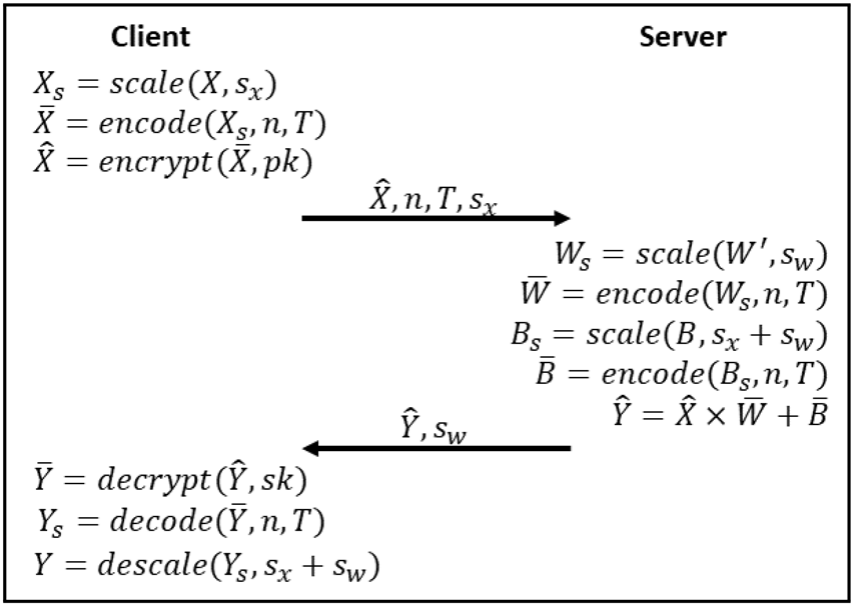
Overview of proposed inference protocol. $\bar{\cdot }$ represents encoded data, while $\widehat{\cdot }$ denotes encrypted data.

Figure 1 describes our threat model where the the patient data, during inference, is encrypted to protect the privacy of a patient, i.e. encrypted genomic data is sent to the cloud, cloud computes on encrypted genomic data homomorphically, and sends back encrypted results. (details in Supplementary). Figure 2 shows the overview of our inference protocol. It consists of input encoding and encryption, weight and bias encoding, computation, decryption, and decoding. The client starts with a $|X| \times f$ matrix *X* containing the input values represented with double-precision floating-point numbers, where |*X*| is the number of inputs and *f* is the number of features. The values of matrix *X* are multiplied by a scaling factor $2^{s_x}$ in order to be converted into integers, a requirement of the BFV encryption scheme^16^ (details on homomorphic encryption in Supplementary). This effectively converts our HE operations into fixed-point arithmetic. The scaled matrix of inputs $X_s$ is then encoded into a matrix of polynomial plaintexts $\bar{X}$, where each polynomial contains *n* coefficients. We pack *n* features of each row into a polynomial. This leads to an encoded matrix of dimensions $|X| \times \lceil f/n\rceil$. It is worth noting that our model requires more precision than what can be represented in the plaintext modulus *t*. From experimental results, we determined that our inputs and weights require 14 bits of precision. Since our inputs are in the interval $0 \le x < 2^8$, we set $s_x = 6$ to represent the inputs in 14 bits. Meanwhile, weights are in the interval $0 \le w < 1$, which leads to $s_w = 14$. Due to the fixed-point arithmetic, the biases must be scaled by $2^{s_x+s_w}$. After the computation, the client will receive outputs that are scaled by a factor of $2^{s_x+s_w}$, like the biases, but that require $8 + s_x + s_w + \lceil \log _{2}(f)\rceil$ bits of precision for representation. For $f = 40,960$, that translates to 44 bits. This is above of what a secure BFV ciphertext with enough noise budget for our computation can support. To cope with that without hindering the accuracy of our model, we used the Chinese Remainder Theorem (CRT) to break our plaintext into a pair of smaller plaintexts, each one under its own modulus. We define our plaintext moduli $T = \{t_0, t_1\}$ as $t_0 = 1,073,872,897$, which provides 30 bits of precision, and $t_1 = 114,689$, offering 16 bits. This means that for every *n* features encoded into a polynomial, we are actually encoding into a pair of polynomials, one with coefficient modulus $t_0$, and another with coefficient modulus $t_1$. For simplicity, we refer to this pair as plaintext polynomial.

The encoded matrix of inputs $\bar{X}$ is then encrypted with the client’s public key *pk*. The encrypted matrix $\hat{X}$ is sent to the server together with public values $\{n, T, s_x\}$. Afterwards, the server scales and encodes the transpose of the matrix of weights *W*, which has dimensions $f \times |Y|$, where |*Y*| is the number of outputs. The transposition is a requirement of our computation. It packs several feature weights of an output in a plaintext polynomial, leading to an encoded matrix of weights $\bar{W}$ of dimensions $|Y| \times \lceil f/n\rceil$. Biases are encoded differently, each bias is encoded into a plaintext polynomial, filling all slots with its value. Finally, the server performs the matrix multiplication of encrypted inputs by encoded weights followed by addition of encoded biases $\hat{Y} = \hat{X} \times \bar{W} + \bar{B}$. The resulting matrix $\hat{Y}$ is returned to the client together with public value $s_w$. The client simply decrypts, decodes, and descale $\hat{Y}$ with its secret key *sk* and obtains the result of the inference in plaintext.

#### Matrix multiplication algorithm

Our privacy-preserving matrix multiplication algorithm, optimized for implementation in HE, is displayed in Algorithm 1. It receives three arguments: The encrypted matrix of inputs $\hat{X}$, encoded matrix of weights $\bar{W}$, and polynomial degree *n*. Each row of $\hat{X}$ represents an input, while each row of $\bar{W}$ represents one output. The computation of the dot product for each input is independent, making this algorithm highly parallelizable. We start the dot product by performing the column-wise multiplication of each row of $\hat{X}$ with the each row of $\bar{W}$ and append the result for each row-row pair into a vector (lines 5–11). Next, we add together all elements of the resulting vector (line 13) and execute $\log _2{n}$ ciphertext rotations and additions to finalize the dot product (lines 14–17). This results in a ciphertext where all its slots contain the result of the dot product of the row–row pair. In order to save memory and reduce communication time, we aim at packing several dot product results into a single ciphertext. For this, first we need to clear the ciphertext slots in all but one carefully chosen position. We do it by multiplying the resulting ciphertext $\hat{c}$ by a plaintext polynomial $\bar{p}$ with one at that specific position and zero in the remaining slots (lines 18–22). Finally, we can compress the dot product results $\hat{R_0}$ by adding them together (lines 25–42). If there are more dot product results than slots in a ciphertext, i.e., $|\hat{X}| \cdot |\bar{W}| > n$, then ciphertexts are appended to the output vector $\hat{Y}$. Lastly, we return the result $\hat{Y}$ (line 43). We provide the mathematical representation of the algorithm in the Supplementary.

#### Approximation of non-linear function in private inference

Tumor prediction is a classification problem which we address using multinomial Logistic Regression (LR). An LR model is trained by reducing the logistic loss function. During inference, the probability that an input $(x \in \mathbb {R}^{1\times d})$, with *d* features, belongs to a class (*k*) is given by $P(y=k|x) = \frac{e^z_k}{\sum ^K_{l=1} e^z_l}$ where $z=Wx+b$, $W \in \mathbb {R}^{K\times d}$ is the weight matrix, and $b \in \mathbb {R}^{d}$ is the bias. The predicted class $(k_p)$ is the class with the highest probability, i.e. $k_p=argmax({P(y=k_i|x)})$ where $k_i \in K$. This non-linear logistic function is computationally expensive in HE; thus, we perform the following approximation for building an ML model that can be used for encrypted inference. Since the logistic function is a monotonically increasing function, we can say that $P(y=k|x)$ for a class is higher if $z_k$ is higher, and since the predicted label depends on the relative probability values, the predicted label can also be calculated using $argmax({z_{k_i}})$. Therefore, during inference, a test input $(x_{test})$ needs to be multiplied with the weight matrix to get the final prediction, i.e. the predicted class $k_p = argmax(W \times x_{test}+b)$. Effectively, for efficient inference, the matrix multiplication between the test inputs and the weight matrix must be fast.

The evaluation of private cancer prediction methodology is dependent on the precision of the plaintext model and performance of the private inference protocol. We report the evaluation of somatic mutation encoding towards accurate cancer prediction in plaintext in “Evaluation of plaintext cancer prediction” section and the performance of the ML model (based on our matrix multiplication methodology) in “Privacy-preserving model evaluation” section.

## Results

Comparison to the state-of-the-art: To the best of our knowledge cancer prediction performance using the exact same dataset has not been published. Therefore, we compare the performance of our plaintext model (accuracy) with the 2019 study on prediction of cancer types since the authors use the same TCGA database^15^. A similar study from 2016^14^ also developed cancer prediction models based on TCGA database but the 2019 study supersedes the former in performance. For privacy preserving implementation of cancer prediction, although the plaintext matrices have thousands of features, they get packed in just a handful of ciphertext (e.g. 40,960 features are encrypted with 5 ciphertexts since each ciphertext can pack n plaintexts, where n is the polynomial degree). Thus, the matrix multiplication algorithm multiplies small matrices. While there are matrix multiplication algorithms with lower complexity than the standard, their benefit is only for large matrices. For smaller matrices they are worse than the naive matrix multiplication due to costs to prepare the matrices. Furthermore, techniques that improve cache utilization in matrix multiplication do not work for HE because ciphertexts are very large, which precludes keeping many ciphertexts in the cache memory. Other encrypted matrix protocols require back-and-forth communication between the client and the server, which is a different approach from ours (send once/receive once) and therefore, not consistent with our usage model, since communication costs are the bottleneck in these protocols^23^. Thus, we implement the standard LR in HE and compare our optimized ML model with it in the encrypted domain.

### Evaluation of plaintext cancer prediction

#### Using only presence of an SNV on a gene

**Figure 3:**
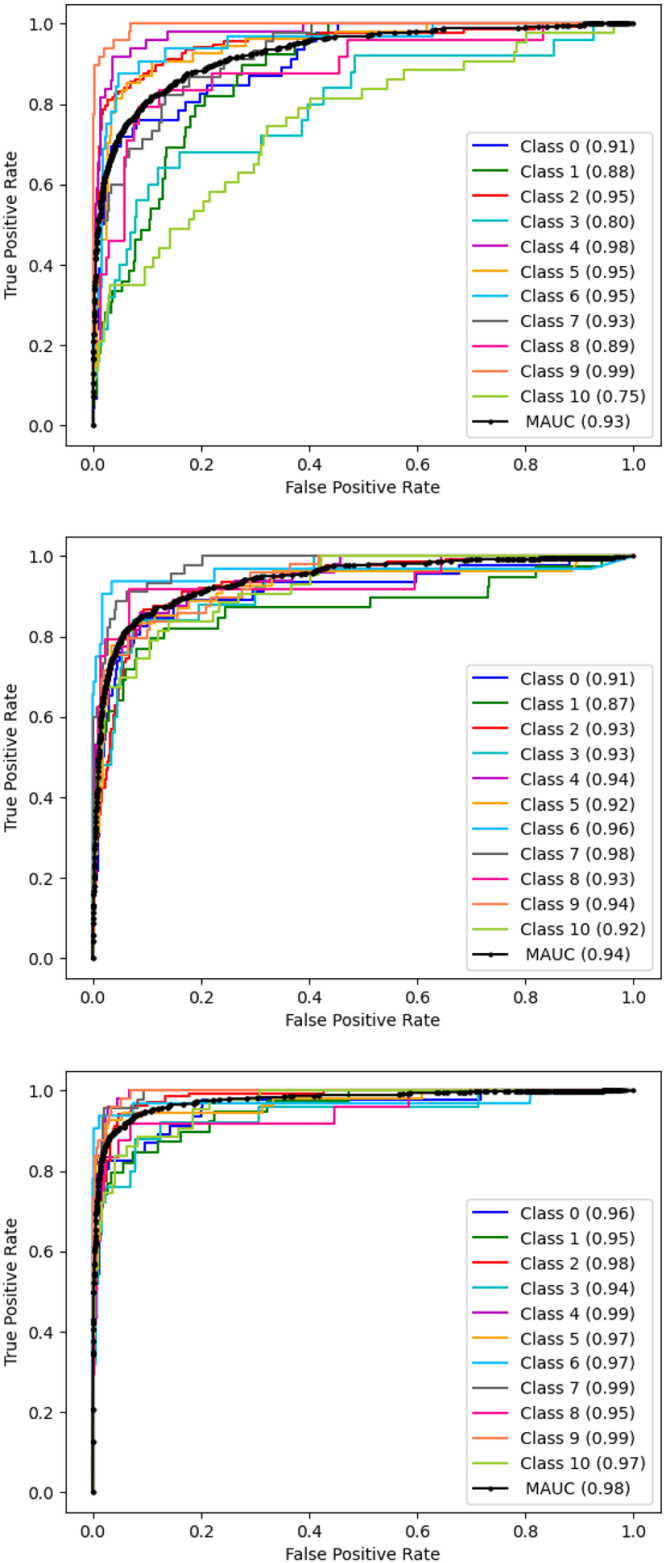
We report the ROC curves and the micro-average scores for the best performing models using different genetic information: (**a**) Using the presence of mutation in a gene as a feature for top 15,000 genes, (**b**) using CNV information in a gene as a feature for top 17,000 genes, (**c**) using both encoded SNV and CNV features with top 34,000 features.

We report different performance metrics and the information on the features used for different models tested in Table 1. We observe that our model achieves a test accuracy of 66.85% and a micro-average area under curve of 0.928 with top 15,000 features. We also plot an Receiver Operating Characteristics (ROC) curve (Fig. 3) for each class and observe that skin cancer (class 9) detection has the highest area under the curve of 0.994 while stomach cancer (class 10) prediction has the lowest area under the curve of 0.754. Although on a slightly different dataset, other ML-based cancer prediction achieved a similar test accuracy of 65.5%^14^ and of 70.08%^15^. These methods also used the SNV frequency to prune the number of features.

#### Using only CNV of the genes

We also experiment with just the copy number information for all the 25,128 genes and run a $\chi ^2$ test to select the top genes. We achieve a slightly higher test accuracy of 71.27% with 17,000 features. From Fig. 3, we observe that the micro average area also improves to 0.94 with the lowest area under curve for detection of breast cancer (class 1) at 0.87 (from 0.754). Higher test accuracy and MAUC show that CNVs have more distinguishing power on the type of cancers than SNVs, when considered individually.

#### Final model: using both SNV and CNV information

We select top *f* features using both SNV and CNV information as described above and train several models (best performing models shown in Table 1) to evaluate different machine learning algorithms for the tumor classification task. SVM with linear, rbf, and polynomial kernels achieve the best test accuracy of 68.13%, 64.82%, and 69.98% with 13,000, 37,000, and 34,000 features respectively. Therefore, SVM does not show much improvement from our baseline that used only the presence of SNVs as features. Comparing our model with the state-of-the-art, we observe a $\approx 13\%$ improvement over the state-of-the-art (from 70.08 to 83.61%)^15^. We achieve a test accuracy of 83.61% with 34,000 features thereby also reducing the number of features. We also observe an improvement in the micro average area to 0.976 when compared with models using just CNV or just presence of SNV. ROC for individual classes also have higher scores with the lowest area under curve of 0.94 for cervical cancer (class 3). All the classes achieve an ROC area under curve of more than 0.9. This experiment also shows that although CNVs are more informative, using both CNV and SNVs result in the highest prediction accuracy. We also test our logistic regression model using mutual information and f-score as feature selection methods but we achieve lower test accuracy of 81.95% with 32,000 features, and $82.68\%$ with 40,000 features, respectively (Table 1). We believe that the performance of our model is higher than that of the state-of-the-art^15^ due to many factors contributed by our approach including the feature engineering, variant effect encoding, as well as addition of CNV information to the feature set.

#### Binary models

To compare with the specific models depicted in the state-of-the-art^15^, we built 11 specific models for each cancer type. Although the minimum accuracy for a binary model is lower than the minimum accuracy reported by the authors, we report a higher value in terms of minimum auc achieved by all binary models. We use both CNV and SNV information to select the top 34,000 features. In these experiments, we train 11 different models, where each one detects a particular tumor. We report the performance (test accuracy and micro-average score) in Table 2. We can see that all of the individual models have a test accuracy of more than 90% and a micro-average area under curve of more than 0.97. The best performing classifier is for kidney with a test accuracy of 97.97% and a micro-average score of 0.996. Even the worst performing binary model (for bronchus and lung) has a test accuracy of 91.34% and micro-average score of 0.974.

#### Predictive genes

**Figure 4:**
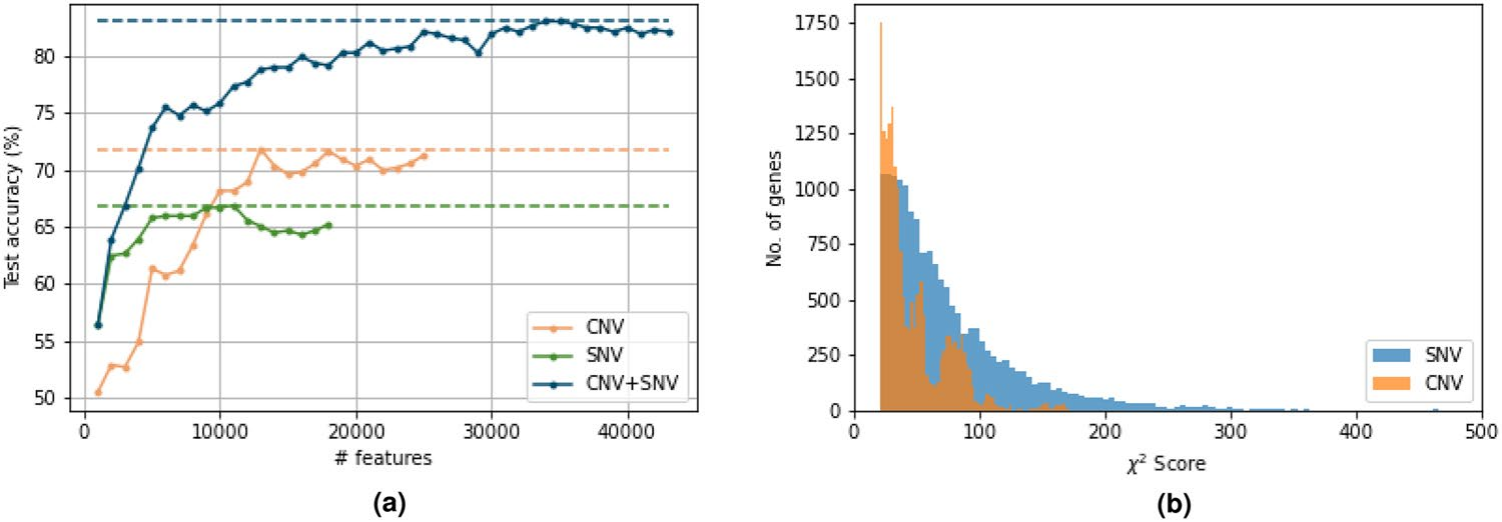
(**a**) Variation of the achieved test accuracy of the trained models with different number of features selected using $\chi ^2$ test. (**b**) The distribution of feature scores for genes contributing CNV and SNV data in the final model.

We discuss our findings on the top genes selected. A total of 17,962 genes are selected for CNV data and 16,038 are selected for SNV data as the most informative features. However, more than 60% of these genes are common, i.e. 11,133 genes are selected based on both CNV and SNV information. Considering the $\chi ^2$ scores, we also observe that the top 1030 genes come from using SNV information. We plot histograms of $\chi ^2$ statistic scores in Fig. 4b and observe that the genes selected based on SNVs are more flat i.e. there are more genes with higher scores. We also verified that the highest score of a gene selected based on SNVs is $\approx 10 \times$ higher than the highest value of a gene selected based on CNVs. Therefore, from feature selection perspective using $\chi ^2$ test, SNV data on the selected genes are statistically *more important* than their CNV counterparts. But CNV information improves the test accuracy by adding potentially more relevant biological information.

When we investigated the top 10 most informative genes based on the SNV information, we found “PTEN” are “APC” genes, which are known tumor suppressors; “MUC16” gene, which is a biomarker for ovarian cancer; “ZFHX3” gene, which is implicated in prostate cancer; “CCDC168” gene, which is known to be associated with Prostate Carcinoma and Uterine Body Mixed Cancer. The other 5 genes in this list are also implicated in important cellular activities that could potentially be related to cancer. The first gene in the top 10 most informative genes based on the CNV information is the first ever known tumor suppressor “RB1”. Similarly, “CDKN2A” and “CDKN2B” genes are also known tumor suppressors. “RCBTB2” gene is known to be repressed in prostate cancer. The “CDKN2B-AS1” gene has the silencing power of many other genes in the genome and strongly implicated in various cancer types. The other 5 genes in this list are also implicated in important cellular activities that could potentially be related to cancer. These genes and their corresponding Gene Ontology (GO) term enrichment are depicted in Supplementary.

#### Insights

The first insight is that biological intuition combined with high-dimensional data analysis methods can together achieve high accuracy (MAUC) while reducing the effective number of features. We further validate, using other studies, that our ML model indeed selects features that are biologically relevant. Using domain expertise, our ML model achieved a high performance of 0.98 MAUC with logistic regression ML model.

We further plot the variation of test accuracy (of a model trained using best hyper-parameters) with the number of features in Fig. 4a and observe that the test accuracy clearly rises when the number of features is lower than the number of samples and begins to saturate only after 30 features. From Fig. 4a we see that to achieve a test accuracy of more than 80%, we need at least 20 features, and to achieve the highest test accuracy, we need more than 30 features. Therefore, private genomic analysis is one such application where extremely high-dimensional must be processed. The second insight motivates the development of matrix multiplication algorithm, which when implemented using BFV, can result in fast yet private prediction.

### Privacy-preserving model evaluation

We evaluate our privacy-preserving cancer prediction model on an AMD Ryzen Threadripper 3960X 24-Core Processor with 128 GB RAM using 24 threads running Ubuntu 20.04 LTS. Encryption and computation operations are threaded, while decryption runs on single core. We implement our model using the E3 framework^24^ with the underlying Microsoft SEAL library^25^ and encryption parameters set as: polynomial degree $n = 8192$, and plaintext moduli $t_0 = 1,073,872,897$ and $t_1 = 114,689$, with a required security level of 128-bits. The cancer prediction model is hosted in the server and the client sends the encrypted genomic data to the server. As a use case, we privately compute cancer label for 543 patients, which constitutes 20% of the dataset. We compare our private logistic regression model with private logistic regression model implemented using standard matrix multiplication (dubbed as standard LR). To the best of our knowledge, there is no HE-based implementation of cancer prediction using CNV and SNV features. Hence, we consider the standard LR as our baseline. Please note all private models are implemented using BFV scheme with the E3 framework.

#### Latency

**Figure 5:**
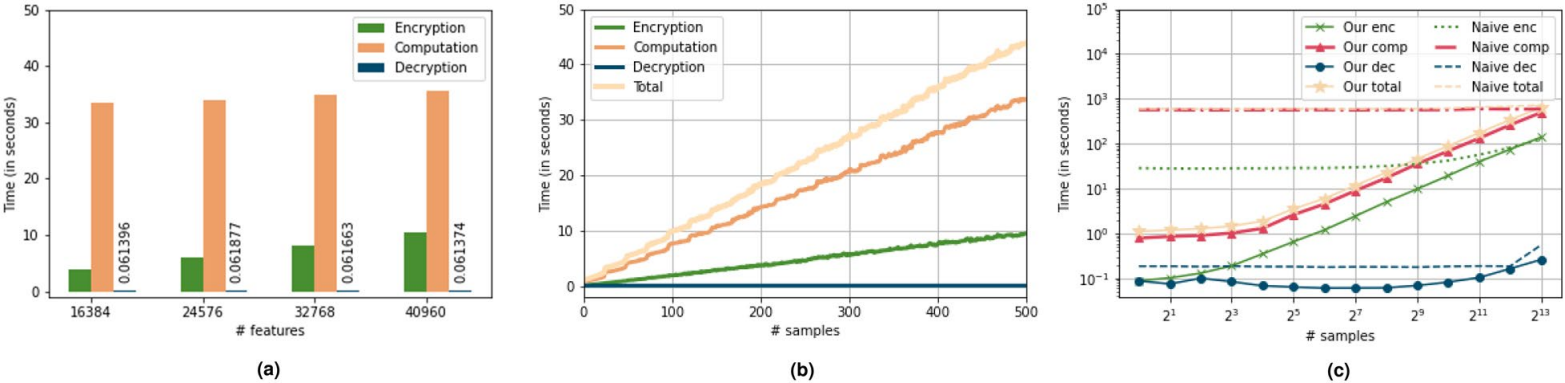
Experimental analysis: (**a**) performance of our private LR-based cancer prediction model as a function of features. (**b**) Timing for different operations as a function of number of test samples. (**c**) Latency comparison of our matrix multiplication algorithm with standard privacy-preserving matrix multiplication.

As mentioned in “Introduction” section private computations using HE are generally designed for high throughput, since popular FHE schemes support batching. For our application, we also prioritize latency, i.e. evaluation of a single sample. We report our findings in Fig. 5b. From the figure we observe that the total amount of time to privately compute cancer label for a sample is 1.08 s and there is a linear increase in time with the number of samples. When the number of samples is low, we notice a constant behavior which may be attributed to the constant costs (like encoding of weights and biases) that become prominent for lower number of samples.

#### Timing evaluation

We report the encryption, decryption, and computation time required for private cancer prediction in Fig. 5a. The time taken to calculate the final cancer label, which is effectively the result of matrix multiplication $(x_{test}W+b)$, is denoted by computation. Computation, understandably, is the most costly operation in private cancer prediction. We observe that even if the number of features increase from 16K to more than 40K ($2.5\times$), the computation time only increases from 33.44 to 35.52 s ($1.06\times$), which corresponds to $\approx 7\%$ increase in test accuracy. Therefore, the matrix multiplication is not the bottleneck for private cancer prediction. The time needed for encryption of the test samples increases with the number of features, with 3.87 seconds for 16K features to 10.40 s for 40K features ($2.68\times$) which indicates a linear increase in the encryption time as a function of number of features. Decryption is the least expensive operation (less than 1 s) as compared to encryption and computation; the values for decryption time are labelled in Fig. 5a. The maximum total time for private inference of the entire test dataset is required when processing 40,960 features, and it is 46.77 seconds.

#### Comparison to standard LR

We compare the performance of our model (i.e., LR with the proposed matrix multiplication) with the LR with standard matrix multiplication while keeping the features, test dataset, and the plaintext methodology same. To study the scaling effect of computational costs with the increment of the number of individuals in the test data (i.e. the number of patients with sensitive data), we generate synthetic data for upto 8192 individuals to represent new individuals since our original test data consists of only 543 individuals.
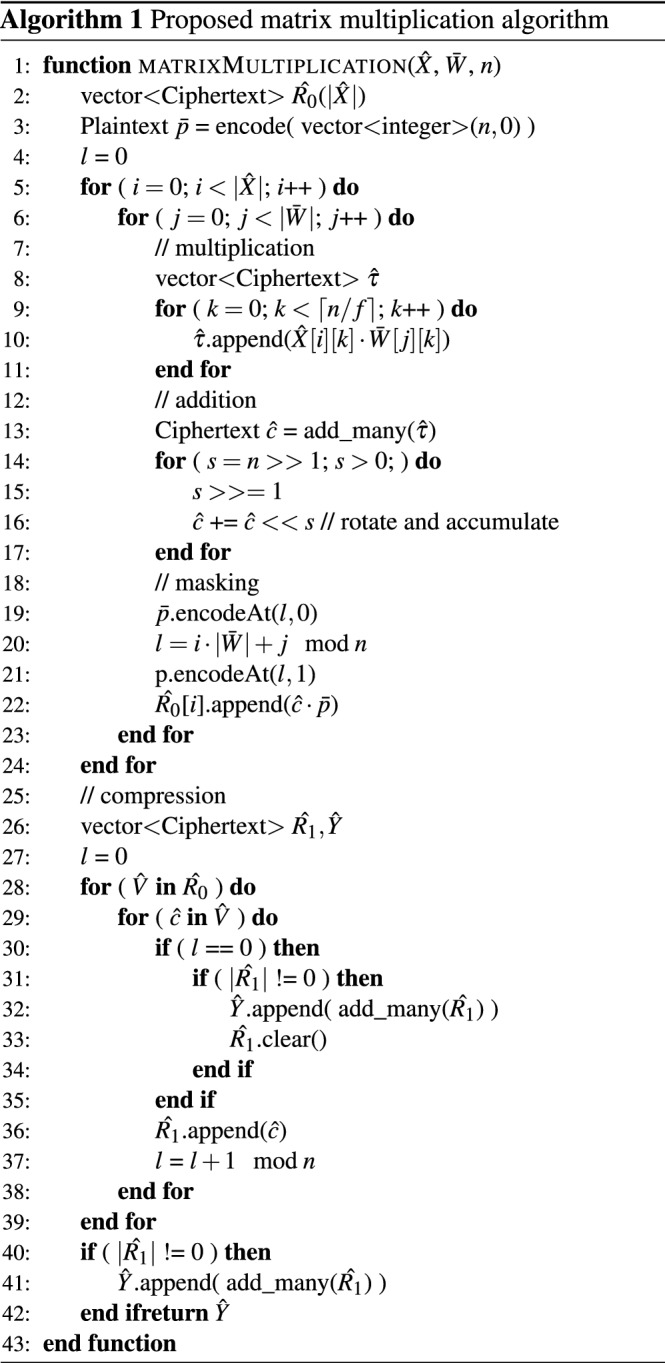

**Table 1:** Performance of cancer prediction model for each for 11 classes. For each machine learning model and feature selection combination, we report the model with highest performance. The best performing model among them, according to test accuracy, is in boldface. Feature selection denotes the statistical feature selection. Accuracy denoted here is the test accuracy after the last fold and the error measure provides the delta change in test accuracy over k-folds. *FS* feature selection, *LR* logistic regression, *SVM* support vector machine, *MI* mutual information.

Feature type	# features	Model type	FS	Accuracy (%)	Error ($\Delta \%$)	MAUC	Precision	Recall	F-score
SNV presence	15K	LR	$\chi ^2$	66.85	4.419	0.928	0.67	0.668	0.66
CNV only	17K	LR	$\chi ^2$	71.27	2.026	0.940	0.718	0.712	0.711
CNV + encoded SNV	13K	SVM (linear)	$\chi ^2$	68.14	0.92	0.942	0.685	0.681	0.68
CNV + encoded SNV	37K	SVM (rbf)	$\chi ^2$	65.01	1.842	0.949	0.6870	0.650	0.637
CNV + encoded SNV	34K	SVM (poly)	$\chi ^2$	69.24	1.11	0.946	0.701	0.692	0.691
CNV + encoded SNV	43K	DNN	$\chi ^2$	61.32	11.602	0.928	0.657	0.613	0.612
CNV + encoded SNV	34K	LR	$\chi ^2$	83.61	1.473	0.976	0.834	0.836	0.834
CNV + encoded SNV	32K	LR	MI	81.95	0.553	0.972	0.822	0.819	0.818
CNV + encoded SNV	40K	LR	f-score	82.68	0.736	0.974	0.827	0.826	0.824

**Table 2:** Performance of individual (binary) models for each cancer type in terms of test accuracy and MAUC.

	Accuracy	MAUC	Precision	Recall	F-score
Class 0 (Bladder)	95.58	0.982	0.952	0.955	0.952
Class 1 (Breast)	94.65	0.983	0.942	0.946	0.944
Class 2 (Bronchus and lung)	91.34	0.974	0.911	0.913	0.912
Class 3 (Cervix uteri)	97.42	0.989	0.972	0.974	0.973
Class 4 (Corpus uteri)	97.42	0.997	0.974	0.974	0.974
Class 5 (Colon)	96.86	0.991	0.967	0.968	0.968
Class 6 (Kidney)	97.97	0.996	0.979	0.979	0.979
Class 7 (Liver and intrahepatic bile duct)	95.39	0.992	0.952	0.953	0.953
Class 8 (Ovary)	97.60	0.993	0.974	0.976	0.975
Class 9 (Skin)	97.42	0.995	0.973	0.974	0.973
Class 10 (Stomach)	96.13	0.982	0.958	0.961	0.958

We measure time for encryption, computation, decryption operations. We plot the timing results in a log-log graph in Fig. 5c. We observe that the total time required for private inference implemented using standard matrix multiplication for similar number of individuals as the test set is approximately 10 min, approximately $10\times$ more than our methodology. Also, the total time required for private inference on 1 individual is 598.25 seconds (similar time required for thousands of individuals), which is $550\times$ more than the time required by our algorithm (our model requires 1.08 s for the same task). Therefore, as compared to standard matrix multiplication, commonly used for implementation of ML models, our algorithm has lower latency, and higher throughput. Please note here that we selected the polynomial degree to be $2^{13}$ and thus, the graph corresponding to the naive approach remains constant till $2^{13}$ samples. For samples more than the polynomial degree, the execution time will double. Therefore, while our approach scales linearly, the naive approach grows in steps, never converging.

#### Generalizing high-dimensional private inference

Healthcare models are difficult to port trivially across datasets (as discussed in “Introduction” section). Cancer detection ML model is no exception. However, our matrix multiplication algorithm is not dependent on input data or weight values (like quantization-based DNN design techniques^26^) and thus, can be reused for datasets requiring HE-based high-dimensional inference. The transferability of our private inference algorithm across applications is an added advantage.

## Conclusion

Current solutions for HE-based privacy preserving inference suffer from impractical overheads; which are further aggravated when dealing with high-dimensional genomic data. In this work we develop a solution for privacy preserving cancer inference on genomic data. We first leverage biological intuition to structure the mutation data and reduce the dimensionality to a practicable limit. For our privacy preserving ML model, we propose a matrix multiplication algorithm to implement logistic regression model, optimized for high throughput and low latency. Our analysis on a real-world genomic dataset shows that our solution achieves cancer prediction MAUC of 0.98 on test dataset and can be computed on encrypted genomic data at $\approx$ 1 s/patient.

## Supplementary Information

Supplementary Information.

## Data Availability

The datasets analysed in the study are available in the octal-candet repository, https://github.com/momalab/octal-candet.
