# Supplementary material for: Privacy-preserving cancer type prediction with homomorphic encryption
Source: Sci Rep. 2023 Jan 30;13:1661. doi: 10.1038/s41598-023-28481-8 (PMC9886900; doi:10.1038/s41598-023-28481-8)
Supplement: Supplementary file 1 — Supplementary Information. [file 41598_2023_28481_MOESM1_ESM.pdf]

Table S1. Data distribution amongst labels between test and training data for different tumor labels.

Tumor type	Training data (%)	Test data (%)
Bronchusandlung	23.08756	25.2302
Bladder	9.769585	8.471455
Skin	9.447005	9.023941
Cervixuteri	5.714286	4.604052
Kidney	5.391705	5.893186
Ovary	5.852535	4.41989
Colon	9.539171	9.023941
Stomach	9.493088	7.918969
Corpusuteri	7.603687	9.944751
Breast	7.465438	7.18232
Liver and intrahepatic bile ducts	6.635945	8.287293

S1 Supplementary Information

S1.1 Dataset

We use the cancer classification dataset from iDASH 2020 competition Task I[9] that was collected for private tumor classification. This data is curated from a centralized database The Cancer Genome Atlas (TCGA) [27] using patients from 11 different cancer types. TCGA cancer genomics dataset consists of 25 petabytes of data. The data includes clinical data, copy number data, DNA sequencing data, imaging data, DNA methylation, microsatellite instability, miRNA sequencing and expression, and protein expression data. But not all patients/cancer types are characterized by each type of data. Several subsets of TCGA resulted in different types of studies. In our work, we study the impact of somatic mutations on prediction of cancer. Our dataset consists of two types of somatic alteration information (considered as two subsets of features): Single-Nucleotide Variations (SNVs) and Copy Number Variations (CNVs) on protein-coding genes. In the SNV subset, four different characteristics are given for each somatic SNV of a gene. These characteristics represent the chromosome location, denotes whether the mutation is a single-nucleotide polymorphism, and the effect of the mutation (using two different measures). The effect of the mutation is calculated using Ensembl Variant Effect Predictor (VEP)[20] and is reported in two ways: 1) A mutation can be considered as one of the following categorical values; high, moderate, modifier, and low, followed by a real number denoting the impact of the mutation. 2) A mutation can qualitatively be denoted as tolerated or deleterious, based on Sorting Intolerant from Tolerant (SIFT) pathogenicity prediction. All of this information reflects the importance of a mutation, i.e. VEP scores help transform an observation of a mutation to its possible impact in development of the tumor. VEP scores help in developing the biological intuition for our feature engineering methodology, which is required as this subset of SNV features contains 2,044,328 somatic mutation rows. In the copy number subset, each gene for each sample (patient) is given a copy number value depending on whether there has been a change from their parents' genes: 0 for no alteration, 1 or 2 for duplication, and -1 or -2 for deletion from one or both the parents, respectively. For each sample, there are 25,128 genes, and thus, 25,128 features. The dataset comes from 2713 patients belonging to 11 different cancer types. The composition of dataset is depicted in Table S1.

S1.2 Grid search for hyper-parameters/model

We performed a grid search over the following classifiers, with their respective hyper-parameters and we report the a subset of models (best-performing models) in Table 1.

Table S2. Grid search amongst classifiers for finding the best model with hyper parameters.

Model	Hyperparameters	Further exploration
SGD Classifier	Ridge penalty, hinge loss, maximum iterations for training: 1000	
Multinomial Naive Bayes	Additive smoothing parameter = 1.0	
Random Forest Classifier	criterion: maximum gini impurity decrease, number of trees = 100	
Decision Tree Classifier	Criterion: maximum gini impurity decrease	
LightGBM classifier	Gradient boosting decision tree, number of trees = 100, learning rate = 0.1	Grid search with selecting features in intervals of 1000 with mutual information and chi-square statistic
Logistic regression	Lasso penalty, liblinear solver, max iterations for training: 10000, balanced class weight initialization)	raw data and positive representation of data
	Ridge penalty,liblinear solver, max iterations for training: 1000)	
	Ridge penalty,Netwon-CG solver, max iterations for training: 1000)	
	Ridge penalty,LBFGS solver, max iterations for training: 1000)	
	Ridge penalty,SAGA solver, max iterations for training: 1000)	
	Ridge penalty, SAG solver', max iterations for training: 1000)	
	Elasticnet penalty,SAGA solver, max iterations for training: 1000)	
	Lasso penalty,liblinear solver, max iterations for training: 1000, balanced class weight initialization)	Grid search with selecting features in intervals of 1000 using chi-squared, mutual information, fscore
Support Vector Machine	gamma: inverse of the radius of influence	raw data and positive representation of data
	gamma = 1/number of features*variance of input, linear kernel)	
	gamma = 1/number of features*variance of input, RBF kernel)	
	gamma = 1/number of features*variance of input, polynomial kernel, degree=5)	
	gamma = 1/number of features*variance of input, sigmoid kernel)	
	gamma = 1/number of features, linear kernel)	
	gamma = 1/number of features, RBF kernel)	
	gamma = 1/number of features, polynomial kernel, degree=5)	
	gamma = 1/number of features, sigmoid kernel)	
	gamma = 1/number of features*variance of input, RBF kernel)	Grid search with selecting features in intervals of 1000 using chi-squared, mutual information, fscore as feature selection metric
Neural Networks	Optimizer:RMSProp Loss:MSE Activation: Softmax	
	Optimizer:Adam Loss:MSE Activation: Softmax	
	Optimizer:Adam Loss:MSE Activation: Softmax	
	Optimizer:Adam Loss:MSE Activation: Softmax	
	Optimizer:SGD Loss:MSE Activation: Softmax	
	Chi-square 8000 features, Optimizer:RMSProp Loss:MSE, Activation: Softmax	
	Mutual information, Optimizer:RMSProp Loss:MSE, Activation: Softmax	
	Mutual information, Optimizer:Adam Loss: CCE, Activation: Softmax	
Best models with different encoding schemes	SNV encoding schemes using both strength and qualitative confidence with different value ranges	

S1.3 Matrix multiplication illustration

Here we describe the matrix multiplication of $Y = \hat{X} \times \bar{W}$, where $\hat{X}$ is the encrypted input matrix (encoded genomic data) and $\bar{W}$ is the encoded matrix of LR weights. The polynomial degree is n, $|X|$ is the number of inputs, $|Y|$ is the number of outputs, and f is the number of features. The operator $\times$ stands for the standard matrix multiplication, while $\otimes$ represents our algorithm, $[\cdot]_n$ is modular reduction over n, and the intervals $[a,b)$ and $[a,b]$ represent elements packed in a ciphertext. When $b < a$, there is a rotation of the n elements of the ciphertext. Function $\rho(\cdot)$ is the element-wise addition of all rotations of a ciphertext, and function $\alpha(\cdot)$ represents the compression part of the algorithm, where one slot of n ciphertexts is selected and combined into a new ciphertext.

$$
Y = \begin{bmatrix} x_{0,0} & \cdots & x_{0,f-1} \\ \vdots & \ddots & \vdots \\ x_{|X|-1,0} & \cdots & x_{|X|-1,f-1} \end{bmatrix} \times \begin{bmatrix} w_{0,0} & \cdots & w_{0,|Y|-1} \\ \vdots & \ddots & \vdots \\ w_{f-1,0} & \cdots & w_{f-1,|Y|-1} \end{bmatrix} =
$$

$$
\begin{bmatrix} x_{0,0} & \cdots & x_{0,f-1} \\ \vdots & \ddots & \vdots \\ x_{|X|-1,0} & \cdots & x_{|X|-1,f-1} \end{bmatrix} \times \begin{bmatrix} w_{0,0} & \cdots & w_{0,f-1} \\ \vdots & \ddots & \vdots \\ w_{|Y|-1,0} & \cdots & w_{|Y|-1,f-1} \end{bmatrix}^{-1} =
$$

$$
\begin{bmatrix} x_{0,[0,n)} & \cdots & x_{0,[f-n,f)} \\ \vdots & \ddots & \vdots \\ x_{|X|-1,[0,n)} & \cdots & x_{|X|-1,[f-n,f)} \end{bmatrix} \otimes \begin{bmatrix} w_{0,[0,n)} & \cdots & w_{0,[f-n,f)} \\ \vdots & \ddots & \vdots \\ w_{|Y|-1,[0,n)} & \cdots & w_{|Y|-1,[f-n,f)} \end{bmatrix} =
$$

$$
\alpha\left(\rho\left(\begin{bmatrix} \sum_{i=0}^{\lceil f/n \rceil -1} x_{0,[i\cdot n,(i+1)\cdot n)} \cdot w_{0,[i\cdot n,(i+1)\cdot n)} & \cdots & \sum_{i=0}^{\lceil f/n \rceil -1} x_{0,[i\cdot n,(i+1)\cdot n)} \cdot w_{|Y|-1,[i\cdot n,(i+1)\cdot n)} \\ \vdots & \ddots & \vdots \\ \sum_{i=0}^{\lceil f/n \rceil -1} x_{|X|-1,[i\cdot n,(i+1)\cdot n)} \cdot w_{0,[i\cdot n,(i+1)\cdot n)} & \cdots & \sum_{i=0}^{\lceil f/n \rceil -1} x_{|X|-1,[i\cdot n,(i+1)\cdot n)} \cdot w_{|Y|-1,[i\cdot n,(i+1)\cdot n)} \end{bmatrix}\right)\right) =
$$

$$
\alpha\left(\rho\left(\begin{bmatrix} t_{0,[0,n)} & \cdots & t_{0,[|Y|-n,|Y|)} \\ \vdots & \ddots & \vdots \\ t_{|X|-1,[0,n)} & \cdots & t_{|X|-1,[|Y|-n,|Y|)} \end{bmatrix}\right)\right) =
$$

$$
\alpha\left(\begin{bmatrix} \sum_{i=0}^{n-1} t_{0,[i,[n+i]_n]} & \cdots & \sum_{i=0}^{n-1} t_{0,[|Y|-n+i,|Y|-n+[|Y|-1+i]_n]} \\ \vdots & \ddots & \vdots \\ \sum_{i=0}^{n-1} t_{|X|-1,[i,[n+i]_n]} & \cdots & \sum_{i=0}^{n-1} t_{|X|-1,[|Y|-n+i,|Y|-n+[|Y|-1+i]_n]} \end{bmatrix}\right) =
$$

$$
\alpha\left(\begin{bmatrix} u_{0,[0,n)} & \cdots & u_{0,[|Y|-n,|Y|)} \\ \vdots & \ddots & \vdots \\ u_{|X|-1,[0,n)} & \cdots & u_{|X|-1,[|Y|-n,|Y|)} \end{bmatrix}\right) =
$$

$$
\begin{bmatrix} \{u_{0,0}, u_{0,n}, u_{0,2n}, \ldots, u_{0,(n-1)\cdot n}\} & \cdots & \{u_{0,|Y|-(n-1)\cdot n-1}, \ldots, u_{0,2n-1}, u_{0,|Y|-n-1}, u_{0,|Y|-1}\} \\ \vdots & \ddots & \vdots \\ \{u_{|X|-1,0}, u_{|X|-1,n}, \ldots, u_{|X|-1,(n-1)\cdot n}\} & \cdots & \{u_{|X|-1,|Y|-(n-1)\cdot n-1}, \ldots, u_{|X|-1,|Y|-n-1}, u_{|X|-1,|Y|-1}\} \end{bmatrix} =
$$

$$
\begin{bmatrix} y_{0,[0,n)} & \cdots & y_{0,[|Y|-n,|Y|)} \\ \vdots & \ddots & \vdots \\ y_{|X|-1,[0,n)} & \cdots & y_{|X|-1,[|Y|-n,|Y|)} \end{bmatrix}
$$

S1.4 Predictive genes analysis

This subsection depicts the top genes selected from the CNV and SNV pool their corresponding Gene Ontology (GO) enrichment terms.

Table S3. Genes selected by our model. The left column denotes the selected genes and the right column represents their GO enrichment terms.

**CNV-based top 10 genes**	**GO enrichment analysis**
RB1	DNA-binding transcription factor activity and enzyme binding
CDKN2A	transcription factor binding
LINC00441	NA
DGKH	NAD+ kinase activity and diacylglycerol kinase activity
RCBTB2	Ran guanyl-nucleotide exchange factor activity
CDKN2B-AS1	NA & Intracranial Aneurysm and Periodontitis
LPAR6	G protein-coupled receptor activity
AKAP11	protein kinase A binding and protein phosphatase1 binding
CDKN2B	protein kinase binding and cyclin-dependent protein serine threonine kinase inhibitor activity
ITM2B	amyloid-beta binding
**SNV-based top 10 genes**	**GO enrichment analysis**
TTN	nucleic acid binding and identical protein binding
PTEN	protein kinase binding and magnesium ion binding
APC	microtubule binding
MUC16	metabolism
DST	calcium ion binding and actin binding
ZFHX3	nucleic acid binding and sequence-specific DNA binding
CCDC168	NA
ATRX	chromatin binding and helicase activity
DNAH5	ATPase activity and microtubule motor activity
PIK3R1	GTP binding and transcription factor binding

S1.5 Homomorphic encryption

Homomorphic Encryption (HE) is a type of encryption that allows for computation on encrypted data without decryption. Let us consider a function, $f(.)$ operating on plaintext operands p_1, p_2, and the equivalent function $f_{enc}(.)$ operating on the corresponding ciphertexts c_1, c_2, such that $c1 = Enc(p_1)$, and $c2 = Enc(p_2)$, where $Enc(.)$ is the encryption function. Then, the computation of the function $f(.)$ on plaintext operands p_1, p_2 is the decryption of computation of the function f_{enc} on ciphertexts, i.e. using HE, we can say that $f(p1, p2) = Dec(f_{enc}(c_1, c_2))$, where $Dec(.)$ is the decryption function. Depending on the type of computation possible on the encrypted domain, there are several types of HE schemes.

For linear models with unencrypted weights, Partial Homomorphic Encryption (PHE) schemes like Paillier[29] can be used. Nevertheless, encryption and decryption operations, which consist of modular exponentiations, hinders the performance of ML models with larger inputs or outputs. In addition, although it is possible to encode several plaintext into a ciphertext in Paillier for certain applications, the density of plaintexts per ciphertext is much lower than in Somewhat Homomorphic Encryption (SHE) or Fully Homomorphic Encryption (FHE). Furthermore, Paillier is not post-quantum secure since it can be broken by Shor's algorithm[28]. Thus, it is not suitable for handling genomics data, since they must be secure for decades or even generations.

A better approach comes from using SHE/FHE schemes like BFV (Brakerski/Fan-Vercauteren)[16] or CKKS (Cheon, Kim, Kim, Song)[30]. CKKS enables fixed-point arithmetic and it is the standard choice for ML applications. During computation, CKKS drops the lower bits of the plaintext after each operation, reducing the precision of the result. With current HE libraries and standard encryption parameters, unfortunately CKKS does not provide enough precision for our model. Conversely, BFV works on integers (modular arithmetic), where we can emulate fixed-point arithmetic by scaling up the double-precision floating-point number into integers. Similarly to CKKS, there is a limitation on how much precision a BFV ciphertext can provide. However, since it computes on modular arithmetic, we can use the Chinese Remainder Theorem (CRT) to break our values into several smaller values, each one under unique modulus coprime to all other moduli. Each smaller value is then encrypted under a different key. In our threat model, thus, the training is not privacy-preserving, but the inference is private. To make inference private we resort to encrypted computation (cancer prediction) using homomorphic encryption. Fig. 1 summarizes our threat model.
